# Brazilian Adults’ Hope and Spirituality in Preoperative Heart Surgery: A Cross-Sectional Study

**DOI:** 10.21470/1678-9741-2022-0230

**Published:** 2023-06-14

**Authors:** Juciano Augusto da Silva Negré, Leonardo Santos de Souza, Elaine Cristina Negri

**Affiliations:** 1 Department of Intensive Care, Hospital Augusto de Oliveira Camargo - HAOC, Indaiatuba, São Paulo, Brazil; 2 Department of Nursing, Universidade do Oeste Paulista - UNOESTE, Presidente Prudente, São Paulo, Brazil; 3 Department of Psychology, Universidade do Oeste Paulista - UNOESTE, Presidente Prudente, São Paulo, Brazil; 4 Department of Psychology, Hospital do Coração de São Paulo - HCor, São Paulo, São Paulo, Brazil; 5 Department of Nursing, Universidade de São Paulo - USP, Ribeirão Preto, São Paulo, Brazil

**Keywords:** Hope, Religion, Thoracic Surgery, Cardiovascular Diseases, Spirituality, Cardiology

## Abstract

**Introduction:**

Given the incipience of domestic studies on hope and spirituality in cardiology, this study evaluated adult cardiac patients’ hope in the preoperative period of cardiac surgery and its potential association with spirituality.

**Methods:**

This is a cross-sectional study carried out at a university hospital in the State of São Paulo (Brazil). A total of 70 patients answered the Herth Hope Scale and a sociodemographic questionnaire before undergoing surgical procedure between January and October 2018. Descriptive and inferential analyses were performed using the Spearman’s rank correlation coefficient and the Mann-Whitney U test. The R-3.4.1 software and SAS System for Windows 9.2 were also used. P-value < 0.05 was considered statistically significant.

**Results:**

Patients had a high prevalence of modifiable risk factors. Having a religion (37.53±4.57) and practicing it (38.79±4.25), regardless of its denomination and time dedicated to that religion, was associated with hope (P<0.01) in the immediate preoperative period of cardiac surgery. However, hope did not exhibit a significant correlation with factors such as age (P=0.09) and time dedicated to religious practice (P=0.07).

**Conclusion:**

Regardless of the religious strand and time dedicated to religious practices as an expression of spirituality, hope was associated with the participants’ religion and religiosity. Considering the importance of this construct on the processes of health and disease, the whole health team should consider in their praxis a setting of conditions to make the patient’s spirituality process feasible during hospitalization.

**Table:** 

Abbreviations, Acronyms & Symbols	
CI	= Confidence interval
CVD	= Cardiovascular disease
DUREL	= Duke University Religion Index
FAITH	= Faith/spiritual beliefs, Application, Influence/importance, Talk/terminal events planning, Help
FICA	= Faith/beliefs, Importance/influence, Community, Action in treatment
HHS	= Herth Hope Scale
HOPE	= Sources of Hope, Religious Organization, Personal spiritual practices, Effects on treatment
ICU	= Intensive care unit
SBC	= Sociedade Brasileira de Cardiologia

## INTRODUCTION

Cardiac surgery represents an alternative for the restoration of the cardiovascular function and quality of life^[1]^ of patients based on the correction, reconstruction, or replacement of affected areas^[2]^ due to the aggravation of cardiovascular diseases, resulting mainly from modifiable risk factors such as smoking, hypercholesterolemia, diabetes, arterial hypertension, abdominal obesity, sedentary lifestyle, inadequate diet, stress, anxiety, depression^[3-5]^, sleep-related problems, and low educational and socioeconomic levels^[4-6]^.

In view of the uncertainty of surgical success and risk of death, many patients use spirituality, religiosity, and hope as possible resources for coping psychologically with this adverse time^[7]^. It should be noted that spirituality is a dynamic subjective process in which individuals seek meaning, purpose, connection, and meaning of life, which can transcend the usual logic or be grounded on humanism in the construction of their personal values, without necessarily involving an individual or community religious practice^[8,9]^.

Religion, on the other hand, refers to an organized system of beliefs, philosophical assumptions, rituals, and ceremonies practiced in public or private contexts, which aim to connect the individual with the divine or transcendent domain while valuing a specific set of individual, collective, and institutional behaviors^[4,9]^; religious behavior or religiosity corresponds to how much the individual believes in, follows, and practices a religion through individual or collective activities, whether in the religious institution or outside it^[9]^.

In contrast, hope and spirituality do not have a universal definition as constructs, even though different authors agree that, as a multidimensional phenomenon, they are related to religious and/or spiritual factors that yield a positive guideline for the individual’s future^[10-12]^. They are frequently assessed in cancer patients, in palliative care^[11-13]^, and in positive psychology^[10,14]^.

Evidence available in health psychology and behavioral cardiology about spirituality indicates that spirituality has an independent association with cardiovascular mortality, as anger and hostility are associated with ventricular dysfunction, diabetes, and early subclinical atherosclerotic cardiovascular disease^[15]^. Patients in the preoperative period of cardiac surgery showed higher levels of optimism and hope associated with positive coping resources based on spirituality and religiosity, which in turn was correlated with lower rates of postoperative complications, as well as lower rates of anxiety, stress, and depression^[15,16]^.

Although some studies have associated hope to a better quality of life and satisfaction with life in patients with cardiovascular disease^[17]^, national studies on this construct in connection with cardiology are still incipient^[18]^. Considering that hope has been shown to be an important variable for positive coping in health and disease processes^[5,13,19]^, as well as spirituality and religiosity^[4,15,18,19]^, a new field to be explored^[18]^ by behavioral cardiology is unveiled.

Based on this scenario, the main objective of this study was to investigate patients’ hope in the cardiac surgery preoperative period. As a dependent variable and for an exploratory purpose, the objective was to review potential associations between hope, spirituality, and religious behavior in this audience according to the independent variables age and frequency of religious or spiritual practices, based on the hypothesis of a positive relationship between them as indicated in the literature.

## METHODS

This observational cross-sectional study was approved by the Universidade do Oeste Paulista Research Ethics Committee (CAAE number: 80236817.5.0000.5515). The survey was performed at a reference public university hospital in the countryside of the State of São Paulo (Brazil). We collected data from adult Brazilian individuals with heart disease during the preoperative period of cardiovascular surgery, between January and October 2018.

A signed consent form was obtained from those patients who agreed to participate in the study after being informed of the study objectives that were compliant with resolutions 466/2012 and 510/2016 that approved the guidelines and regulatory standards for human research, and the study followed the Strengthening of the Reporting of Observational Studies in Epidemiology (or STROBE) guidelines for reporting observational studies^[20]^.

**Fig. 1:**
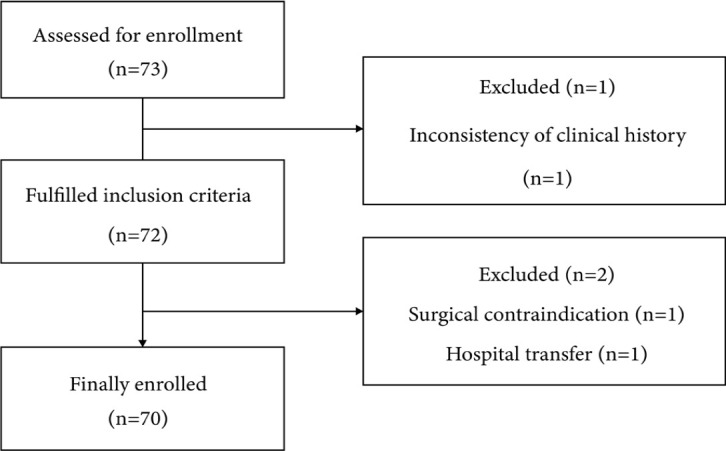
Flowchart of patients in the study.

The selection of participants was non-probabilistic and for convenience. We recruited 73 elective admission patients aged 18 years or older, who received a confirmed cardiac diagnosis from the multidisciplinary medical record and imaging exams, who were in the immediate preoperative period (24 hours before the procedure), and who did not have cognitive or psychiatric impairment that would impair the understanding of the surgical procedure. After applying the eligibility criteria, three potential participants were excluded, as shown in Figure 1.

Patients were instructed on admission by the nurse on duty about the routine of the unit, family visits, medical records, and the right of visit of the religious leader indicated by the patient, according to his/her desire and request, or by the patient’s companion; those are not data quantified by the investigators due to the free access granted to accredited religious leaders to the hospital.

As an institutional routine, in the preoperative period, patients received a daily multiprofessional visit at bedside and a clinical evaluation carried out by a clinical cardiologist, cardiac surgeon, anesthesiologist, nurse, psychologist, and physical therapist, being informed at this time about the anesthetic-surgical benefits and risks based on comorbidities and the diagnosis severity, need for mechanical ventilation, intensive support, and scheduled date for the procedure and rehabilitation, followed by clarification of doubts.

Preoperative care complies with the Brazilian Guidelines for Cardiovascular and Perioperative Assessment, involving review of the electrocardiogram and echocardiogram, cardiac catheterization and angiography, hematological and biochemical tests, suspension of anticoagulants, antiplatelet agents, and other medications necessary according to the drug’s duration of action, control of perioperative and postoperative coagulogram, in addition to blood reserve. Whenever there were no significant interactions with anesthetics, chronic medications such as hypoglycemics, corticosteroids, and psychotropics were maintained until the morning of the surgery and restarted in the immediate postoperative period.

Midazolam was used as preanesthetic medication, and the anesthetic technique consisted of general anesthesia induced with balanced fentanyl, with the use of inhalant and venous agents such as benzodiazepines, opioids, and neuromuscular blockers. The cardiac surgical technique employed was sternotomy with extracorporeal circulation. At the end of the surgery, hemodynamically stable patients were extubated in the operating room after neuromuscular blockade reversal, and unstable patients were extubated within the first six hours in the coronary intensive care unit (ICU). Postoperative analgesia was based on non-steroidal anti-inflammatory drugs and intravenous opioids. Delirium conditions were treated with antipsychotics, benzodiazepines, environmental management, and cognitive stimulation.

Since the study was developed before the coronavirus disease 2019 (or COVID-19) pandemic, patients, throughout their stay in the ward, were entitled to a 24-hour companion and family visit once a day. On the other hand, during their stay in the coronary ICU, which lasted five days on average, the visit of up to two family members per day for one hour was allowed at the same time of the medical report elaboration since a patient’s companion in the unit was not allowed. Considering the discharge schedule, guidance to the patient and to his/her companion about cardiac and metabolic rehabilitation was enhanced with appropriate referrals to multiprofessional visits. As an institutional code of ethics, it was recommended that the multiprofessional team would not intervene directly on the patient’s spirituality, but that patient’s access to a direct contact with their spiritual and/or religious leader or community should be facilitated.

The patients’ multiprofessional records were the source of all demographic, clinical, and preoperative data. To investigate the perception of the illness process, in connection with the spirituality and religiosity of the Brazilian cultural diversity, a semi-structured questionnaire previously prepared by the authors was used. The objective was to assess the existence and type of relationship with the sacred or the transcendent, the beliefs in the meaning of life, as well as the frequency of individual and collective spiritual or religious activities practiced per month. On the other hand, hope was assessed using the Herth Hope Scale (HHS)^[12]^, a self-report instrument cross-culturally validated for the Brazilian population to measure the individual’s level of hope through 12 items in a four-point Likert-type format. Responses ranged from one (completely disagree) to four (completely agree). The total score ranged from 12 to 48 points, and the higher the score, the higher the level of hope^[12]^.

The instruments were applied individually after the patients read and sign the free and informed consent form in their rooms. The mean time for answering the questionnaires was 10 minutes. After collection, the data were coded and double-typed in Microsoft Excel, version 22, for Windows spreadsheets, in order to review possible discrepancies between the typists. It should be noted that the first author (researcher responsible for conducting the study) blinded the team of typists and statistical analysis as well as the other authors of this study who worked on the interpretation of the results and preparation of the manuscript.

### Statistical Analysis

**Fig. 2:**
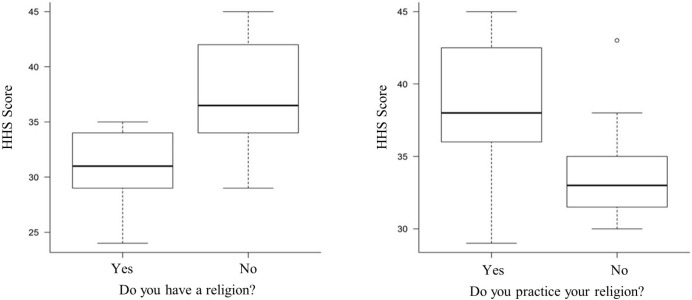
Level of hope between religious and non-religious patients (Herth Hope Scale [HHS]). At least 75% of the patients who stated having a religion and practiced it had a score ≥ 34 points on the HHS.

The characterization of the participants was set based on absolute and relative frequencies for the categorical variables and mean ± standard deviation for numerical variables. In order to identify possible associations between HHS, age, and frequency of religious practice, the Spearman’s rank correlation coefficient (*Ρ*) was used. To compare groups (religious and non-religious) in relation to the level of hope, the non-parametric Mann-Whitney U test was used. The graphics presented (Figure 2) were developed with the help of the R-3.4.1 software and the analyses were carried out using the SAS System for Windows 9.2, adopting a significance level of 5% for all analyses.

## RESULTS

A total of 70 patients were included in the study after establishing the eligibility criteria as illustrated in the flowchart (Figure 1).

The patients’ sociodemographic and clinical characterization showed a predominance of male patients, married, retired, with income between one and three minimum monthly wages, and complete high school; each patient had at least three cardiovascular risk factors prevalence: hypertension, family history, and sedentary lifestyle were corroborating the literature data.

Although patients had health risk behaviors such as alcoholism and smoking, we could perceive a reduction in exposure to these factors over time. In addition, acute myocardial infarction and valve regurgitation were the main diagnoses of patients upon hospital admission and, consequently, myocardial revascularization and valve replacement surgery were the main surgical procedures involving these participants, as shown in Table 1.

**Table 1:** Clinical and sociodemographic characteristics and associations with hope.

Characteristic	Total (N=70)	Mann-Whitney U test
		*P*-value
Age (years)	60.29±12.33	
Male, n (%)	47 (67.14%)	
Married, n (%)	53 (75.71%)	
Widower, n (%)	17 (24.28%)	
Elementary school, n (%)	18 (25.71%)	35.85±4.66 *P*=0.23
High school, n (%)	25 (35.71%)	37.26±4.97
University education, n (%)	14 (20.0%)	36.12±4.71
Professionally active, n (%)	36 (51.42%)	
Unemployed, n (%)	4 (5.71%)	
Retired, n (%)	30 (42.85%)	
Income up to 1 monthly minimum wage^*^, n (%)	12 (17.14%)	35.4±5.84 *P*=0.37
Between 1 and 3 minimum wages^*^, n (%)	58 (82.85%)	37.26±4.67
Number of people in the family	3.29±1.35	
Has religion, n (%)	64 (91.42%)	37.53±4.57 *P*≤0.01
Practices religion, n (%)	48/64 (75.0%)	38.79±4.25 *P*≤0.01
Spiritual/religious activity(ies) per month, n (%)	4.38±2.49	
Overall score on the HHS	36.94±4.89	
Coexisting conditions		
Systemic arterial hypertension, n (%)	63 (90.0%)	
Diabetes mellitus, n (%)	27 (38.57%)	
Dyslipidemia, n (%)	23 (32.85%)	
Family history of CVD, n (%)	62 (88.57%)	
Alcoholism, n (%)	15 (21.42%)	
Time of exposure to alcohol (years)	23.13±19.32	
Former smoker, n (%)	31 (44.28%)	
Active smoker, n (%)	9 (12.85%)	
Time of exposure to tobacco (years)	28.19±15.31	
Sedentary lifestyle, n (%)	44 (62.85%)	
Previous cardiac surgery, n (%)	11 (15.71%)	36.18±5.19 *P*=0.43
Admission diagnosis		
Aneurysm, n (%)	6 (8.57%)	34.33±1.21 *P*=0.15
Acute myocardial infarction, n (%)	37 (52.85%)	37.49±5.19 *P*=0.36
Valvular insufficiency, n (%)	22 (31.42%)	37.09±4.88 *P*=0.93
Arterial occlusion, n (%)	5 (7.14%)	34.4±2.7 *P*=0.23
Designated procedure		
Aneurysm repair, n (%)	6 (8.57%)	
Valvuloplasty, n (%)	6 (8.57%)	
Valve replacement, n (%)	2 (2.85%)	
Myocardial revascularization, n (%)	36 (51.42%)	
Revascularization and valve replacement, n (%)	2 (2.85%)	
Valve replacement, n (%)	18 (25.71%)	

* Minimum wage was equivalent to R$937.00 (Brazilian Real) per month at the time of data collection

CVD=cardiovascular disease; HHS=Herth Hope Scale

Still in relation to Table 1, with regard to spirituality, all patients expressed beliefs that positively influenced their individual, interpersonal, or nature connection, mainly based on religion; they identified themselves as Catholics (n=43; 67.2%), Evangelicals (n=19; 29.7%), Umbandaists (n=1; 1.6%), and Buddhist (n=1; 1.6%).

The participants of this study showed high levels of hope based on the raw score obtained in the HHS, and the only variables that showed an association with hope in the cardiac surgery preoperative period based on the Mann-Whitney U test were the expression of spirituality through religion and religiosity, which also resulted in greater variability in the HHS score as shown in Figure 2.

In order to verify the hypothesis about the potential relationship between hope, age group, and frequency of religious practice, the Spearman’s rank correlation test showed that there was no statistically significant relationship between the variables, as shown in Table 2.

**Table 2:** Correlation between hope, age group, and religious practice.

Spearman’s rank correlation						
Variables		N	Coefficient	95% CI		*P*-value
HHS Score	Age (years)	70	-0.21	-0.42	0.03	0.09
	If you practice a religion, how often do you do it? (monthly)	48	0.26	-0.03	0.51	0.07

CI=confidence interval; HHS=Herth Hope Scale

## DISCUSSION

This study, which aimed to assess the hope and spirituality of patients who had been designated for heart surgery, found a positive association in the preoperative period between religion, religiosity, and hope in this framework and did not identify significant differences between mean spirituality and hope according to the diagnosis, gender, and history of cardiac surgery, as reported by Bezerra et al.^[18]^, partially confirming our primary hypothesis. Despite the lack of significant difference in hope according to gender, interventional strategies that prioritize the female audience may be relevant considering that the literature reports higher levels of anxiety and depression in this subgroup^[21,22]^.

In addition, variables such as age and amount of time dedicated to religious practice, despite the weak correlation established, were inconclusive in establishing relationships of dependence with hope. It should be noted that the investigation by Bezerra et al.^[18]^ showed weak positive correlations between hope and the variables income (*P*=0.007) and years of study (*P*=0.013), which was not replicated in this study, despite reports in the literature that also support the existence of associations between hope and education^[13]^.

A possible interpretation of these data is that, regardless of the age group or time dedicated to religious activities, the quality of the connection established by patients with religion in order to exercise their spirituality is more valuable than the frequency or method used for this purpose. In this connection, Bezerra et al.^[18]^ did not identify a statically significant relationship between hope and age, even considering that the elderly population showed better scores in connection with religious, spiritual, and total well-being; no difference related to hope was observed between elderly and non-elderly people.

On the other hand, international studies^[16,23]^ have identified relationships between advanced age and spirituality based on religion in the preoperative period, contributing, with other factors, to the development of a predictor of lower levels of anxiety or depression in the postoperative follow-up of cardiac surgery, as well as how religiosity, optimism, and hope were correlated with lower rates of anxiety or depression.

In this connection, positive, optimistic religious behavior, and hope can play a protective role with regard to the mental health of in-patients earmarked for heart surgery^[14,16]^, just like spirituality and hope are correlated with lower levels of cytokines and other inflammatory markers that favor the development of cardiovascular diseases^[4,9,15,24]^. Another cross-sectional Brazilian study^[13]^ also identified associations between religious practice and higher levels of hope in HHS, besides a negative correlation with depressive symptoms in its sample of cancer patients undergoing chemotherapy treatment.

The importance of spirituality on health and on comprehensive and patient-centered care is associated with cardiovascular diseases based on modifiable factors^[4,5]^; the Sociedade Brasileira de Cardiologia (SBC) has recently developed and established recommendations in their Cardiovascular Prevention Guidelines update for evidence-based practices in spirituality^[4]^. In this connection, cardiovascular medicine has increasingly identified the need to break out with the Cartesian vision in order to be able to assess, diagnose, treat, and prevent illness, holistically and comprehensively^[4,15]^.

Based on the recommendations of the SBC (Table 3)^[4]^ on spiritual and health practices, we can legitimize the importance of an agile approach to the screening or anamnesis of spirituality and religiosity, especially considering chronic diseases and poor prognoses, given its safety and usefulness, as well as welcoming, complying with and providing specialized support to patients in spiritual suffering by promoting subjective well-being and reducing mortality. The recommendations on other behaviors involving intervention on spirituality are still discordant, with the exception of religious prescription and evaluation of spirituality in critical and acute situations, in which the consensus has been reached that such actions should not be carried out^[4]^.

**Table 3:** Spiritual and health practices - recommendation classes and levels of evidence.

Recommendation	Recommendation class	Evidence level
Brief spirituality and religiosity screening	I	B
Spiritual anamnesis of patients with chronic diseases or poor prognosis	I	B
Respect and support the patient’s personal religions, beliefs, and rituals that are not harmful to treatment	I	C
Support by a trained professional for patients in distress or with spiritual demands	I	C
Organizational religiosity is associated with reduced mortality	I	B
Hospital training program in spirituality and religiosity	IIa	C
Spiritual history of stable or outpatients	IIa	B
DUREL, FICA, HOPE, or FAITH questionnaires to assess spirituality	IIa	B
Meditation, relaxation techniques, and stress relief	IIa	B
Spirituality and religiosity potentially increase survival	Iia	B
Spiritual empowerment techniques such as forgiveness, gratitude, and resilience	Iib	C
Assess spirituality and religiosity in patients in acute and unstable situations	III	C
Prescribing prayers, religious practices, or specific religious denomination	III	C
Adapted from Précoma DB et al., 2019^[4]^		

DUREL=Duke University Religion Index; FAITH=Faith/spiritual beliefs, Application, Influence/importance, Talk/terminal events planning, Help; FICA=Faith/beliefs, Importance/influence, Community, Action in treatment; HOPE=Sources of Hope, Religious Organization, Personal spiritual practices, Effects on treatment

Although few studies have investigated hope in the cardiac surgery preoperative period^[18]^, the results of this work, which are in line with the literature, suggest that the provision of conditions for the patient to experience his/her spirituality can favor an adaptive coping based on hope and optimism, contributing to the reduction of stress, anxiety, depressive symptoms, and mortality^[2,4,8,9,13-16,18,19,23]^, because when an adequate environment is provided for the expression of spirituality, the understanding of risks and benefits of the surgical procedures as well as adherence to medications^[4,19]^ and non-drug treatments are observed^[4,8,9,24]^.

Considering that spirituality and religiosity seem to exert control over health risk behaviors^[4,8,9,14]^, evaluation of the effectiveness of the support and training programs in spirituality for health professionals becomes essential, since their approach can be performed quickly by any trained health professional^[9]^, without affecting the time available for the administration of other care, thus improving the quality of transdisciplinary patient-centered care^[4,8]^.

### Limitations

The limitations of this study are related to the survey design and sample size, which interfere with the validity of the results obtained for populations in large urban centers or in supplementary health care settings. In this connection, prospective and multicenter observational studies with more robust samples can support data for experimental studies that can assess the effects of interventions based on spirituality and religiosity, in addition to understanding their apparent relationship with hope, considering that patients who manifest an active religious behavior have higher levels of hope compared to other patients^[13,19]^.

## CONCLUSION

Even if the care team did not adopt direct religious measures in the care process, it is possible that indirectly its intention to alleviate suffering and ensure comfort, associated with the promotion of spaces for contact with a religious leader or community and spiritual practices during visits by family members or individually, have positively influenced the results of hope in the cardiac surgery preoperative period, as well as the effective communication with the patient and his family during the multiprofessional visits and the daily medical report visit that favors a therapeutic relationship of greater trust.

In this sense, it is recommended that surgeons, cardiologists, nurses, psychologists, and other health professionals validate and expand the possibilities of the patient maintaining individual or collective internal or external contacts with their spirituality in the visits, reserve any free time available in the care routine throughout hospitalization so that the patient can meditate, pray, reflect, read, watch, listen to spiritual or religious content if desired, with fewer interruptions, as well as receive religious and family visits, enabling a collective exercise of spirituality whenever possible with the reference people of the patient’s social cycle. Another possibility is to provide a specialized chaplaincy service trained for a comprehensive approach including an approach that takes into account the cultural and regional context and the patient’s individual spiritual support needs in order to contribute as a psychosocial protection factor to cope with hospitalization.

It is concluded that there was a positive relationship between hope and the exercise of spirituality through religiosity in the preoperative period of cardiac surgery in the participants of this study. This relationship needs to be further explored in the literature due to the religious diversity in Brazil and the difficulties in the standardized measurement of spirituality, although the consensus on patient-centered care supports the evaluation and promotion of conditions that stimulate hope based on spirituality and religiosity as an adaptive coping tool in the framework of illness, hospitalization, and perioperative period.

**Table:** 

Authors’ Roles & Responsibilities	
JASN	Substantial contributions to the conception or design of the work; or the acquisition, analysis, or interpretation of data for the work; drafting the work or revising it critically for important intellectual content; agreement to be accountable for all aspects of the work in ensuring that questions related to the accuracy or integrity of any part of the work are appropriately investigated and resolved; final approval of the version to be published
LSS	Substantial contributions to the conception or design of the work; or the acquisition, analysis, or interpretation of data for the work; drafting the work or revising it critically for important intellectual content; final approval of the version to be published
ECN	Substantial contributions to the conception or design of the work; or the acquisition, analysis, or interpretation of data for the work; drafting the work or revising it critically for important intellectual content; final approval of the version to be published
